# Precipitation and wood type determines stem and soil greenhouse gas fluxes in a subtropical forest

**DOI:** 10.3389/fpls.2026.1753330

**Published:** 2026-03-11

**Authors:** Guanghui Yang, Wanchen Xu, Wanyu Ning, Men Jia, Pei Wang, Yuanqiu Liu, Chunsheng Wu

**Affiliations:** 1 The School of Hydraulic Engineering, Jiangxi University of Water Resources and Electric Power, Nanchang, China; 2 School of Soil and Water Conservation, Jiangxi University of Water Resources and Electric Power, Nanchang, China; 3 Key Laboratory of Silviculture, Co-Innovation Center of Jiangxi Typical Trees Cultivation and Utilization, College of Forestry, Jiangxi Agricultural University, Nanchang, China; 4 Lushan Nature Reserve of Jiangxi, Lushan Mountain National Forest Ecological Station, Jiujiang, China

**Keywords:** carbon dioxide, *Cunninghamia lanceolata*, methane, nitrous oxide, sap flow

## Abstract

Research on greenhouse gas (GHG) fluxes has predominantly focused on subtropical soils, with far less attention given to emissions from tree stems. In particular, year-long simultaneous measurements of both soil and tree stem fluxes in these forests are lacking, and data on standing dead trees is exceptionally scarce. We determined the dynamics of standing dead and live tree stems, and soil CH_4_, N_2_O and CO_2_ fluxes in a subtropical forest. We determined GHG fluxes from standing dead and live tree stems with three different tree heights (10 cm, 50 cm and 150 cm) of *Cunninghamia lanceolata* from January 2023 to December 2024 and subjected to analysis by gas chromatography. Measurements of environmental parameters were conducted in tandem with those of fluxes and xylem sap flow. Live tree stems contributed less to the annual GHG dynamics than standing dead trees. Live and standing dead tree stems generally acted as net annual sources of CH_4_, N_2_O, and CO_2_. Tree stem GHG fluxes decreased with decreasing precipitation. Soil was a sink of CH_4_, but a net CO_2_ and N_2_O source. Isolated emission peaks dominated the temporal dynamics of stem CH_4_, N_2_O, and CO_2_ fluxes and significantly contributed to the net annual fluxes. The CH_4_, N_2_O, and CO_2_ efflux from both live and standing dead tree stems exhibited a similar seasonal trend. The status (live or dead) and height of the trees significantly influenced stem GHG dynamics. During the study, CH_4_ emissions from tree stems (across different heights and precipitation conditions) offset an estimated 55.61~60.03% of the soil’s CH_4_ sink capacity. Here, we demonstrate for the first time a strong correlation between stem greenhouse gas fluxes and sap flow in subtropical forests. The stem fluxes of CH_4_ in live and standing dead trees represented a combination of soil-derived and stem-produced methane, whereas CO_2_ and N_2_O fluxes were primarily soil-derived.

## Introduction

A globally significant portion of the world’s carbon (C) and nitrogen (N) reserves is housed in subtropical soils (Pan et al., 2011). Changes in greenhouse gas [carbon dioxide (CO_2_), methane (CH_4_), and nitrous oxide (N_2_O)] dynamics from the destabilization of these key C and N pools could disrupt local biogeochemical cycles and ultimately affect the global climate. Subtropical forest soils commonly act as a net source for CO_2_ and N_2_O but a sink for atmospheric CH_4_ (Tang et al., 2006; Quebbeman et al., 2022; Cui et al., 2022; Qiu et al., 2024). However, precipitation modification for enhanced forest productivity directly affects soil hydrology (Boisvenue and Running, 2006; Gustafson et al., 2017; Jones et al., 2022; Meli et al., 2024; Jain et al., 2025). This hydrological factor is the primary control on forest greenhouse gas (GHG) balances (Yang et al., 2022; Li et al., 2024; Hao et al., 2025). Aerobic shifts following groundwater table lowering also reshape GHG fluxes in forest soils (Korkiakoski et al., 2019; Pihlatie et al., 2010; Lohila et al., 2011; Hao et al., 2025).

The exchange of CO_2_, CH_4_, and N_2_O gases between tree stems and the atmosphere represents a substantial contribution to the greenhouse gas budget of forest ecosystems (Wang et al., 2019; Barba et al., 2019a; Machacova et al., 2016; Bréchet et al., 2025). The exclusion of stem fluxes from most GHG models and assessments is attributable to their significant spatio-temporal variability, uncertainty, and the complexities involved in ecosystem-level upscaling (Machacova et al., 2019; Barba et al., 2019a, Barba et al., 2019). In addition, most studies on CH_4_ fluxes have been conducted for live tree stems within forests and wetlands (Feng et al., 2022; Zhang et al., 2022; Epron et al., 2023; Jeffrey et al., 2021, Jeffrey et al, 2024; Moisan et al., 2025); few have studied standing dead tree stems in subtropical forests (Hettwer et al., 2025a).

Despite the abundance of long-term studies on seasonal soil GHG flux dynamics, annual-scale measurements of stem fluxes are still notably scarce (Mander et al., 2022; Jeffrey et al., 2023; Machacova et al., 2019; Hao et al., 2025). A characteristic limitation of previous studies is their reliance on short-time measurement periods confined to the growing season (Wen et al., 2017; Barba et al., 2021; Gauci et al., 2010; Moisan et al., 2025). However, a growing body of evidence highlights the significance of stem fluxes not only in winter (Machacova et al., 2019) but also during the autumn and spring seasons (Mander et al., 2021; Yong et al., 2025), which are now recognized as major contributors to cumulative stem N_2_O emissions. Thus, it is essential to advance our knowledge of the seasonal patterns in stem fluxes and the factors that govern them.

A diversity of biophysical mechanisms is responsible for driving GHG production and consumption in soil and stem systems (Moisan et al., 2025). Two key microbial processes regulate CH_4_ levels in forest soils: production under anaerobic conditions (methanogenesis) and consumption under aerobic conditions (methanotrophy) (Ni and Groffman, 2018). The production of N_2_O is primarily linked to microbial denitrification and nitrification (Butterbach-Bahl et al., 2013), whereas CO_2_ emissions are largely attributable to respiration from both plant roots and soil microbes (Jiang et al., 2020). Evidence shows that microbial production of CH_4_ and N_2_O can occur inside of tree stems (Keppler et al., 2006; Gauci et al., 2010; Barba et al., 2019a), as well as on the bark surface for CH_4_ (Lenhart et al., 2015). Soils produce CH_4_ in the anaerobic layers and are transported to the atmosphere through trees by different pathways, including ventilation (Schröder, 1989; Große and Schröder, 1984), diffusion (Megonigal et al., 2020; Pangala et al., 2014, Pangala et al., 2017), and transpiration (Covey and Megonigal, 2019). In addition, the rate of CO_2_ efflux from tree stems is regulated by the interplay of stem photosynthesis and respiration (Salomón et al., 2021; Gansert and Burgdorf, 2005). Therefore, to accurately estimate forest GHG fluxes, the accounting of tree emissions must be included (Liu et al., 2020; Pangala et al., 2013, Pangala et al., 2015; Moisan et al., 2025), especially for standing dead trees (Martinez and Ardón, 2021; Hettwer et al., 2025a).

The exchange of these GHGs between the soil, stems, and the atmosphere is modulated by the interplay of various environmental factors and biological processes. The production and solubility of gases in the soil are regulated by the interplay of several factors, including soil temperature, water table depth (WTD), soil water content (SWC), and nutrient availability (Teskey et al., 2008; Pitz and Megonigal, 2017; Barba et al., 2019a; Moisan et al., 2025). Root system density directly governs the absorption extent of dissolved gases by plant roots (Puhe, 2003). Within-tree gas transport occurs via the xylem, where gases are moved upward with the sap flow as a result of pressure gradients (Gansert and Burgdorf, 2005). The conduits transport of GHG emission are the potential source for standing dead tree stems from forest soils (Gorgolewski, 2022; Hettwer et al., 2025a). Recent evidence indicates axial upward gas diffusion through the bark layers of certain trees, a process that operates independently of the transpiration stream (Jeffrey et al., 2024). The rate and pathway of gas efflux from stems to the atmosphere are strongly influenced by morphological and physiological attributes, such as wood density, bark properties, lenticel abundance, and stem type (Gorgolewski, 2022; Pitz et al., 2018; Pangala et al., 2013; Teskey et al., 2008; Yong et al., 2025).

Given that xylem sap flow is a primary mechanism for gas transport in living tree stems, it becomes essential to determine the extent to which it shapes the final GHG flux (Ranniku et al., 2024). However, direct field evidence linking sap flow to tree stem CH_4_ fluxes is lacking (Takahashi et al., 2022; Hettwer et al., 2025b), and a correlation with N_2_O fluxes has been observed only in peatland (Ranniku et al., 2024) and upland (Barba et al., 2021) forests. The role of internal xylem CO_2_ transport and its diffusive flux to the atmosphere in forest carbon budgets remains unclear and unquantified (Barba et al., 2021; Kunert, 2018).

The vertical profile of tree stem fluxes is valuable for identifying the original sources of the released gases. A primarily soil-derived origin of fluxes is indicated by a vertical trend where the flux magnitude is highest at the stem base and progressively decreases with height (Pitz and Megonigal, 2017; Machacova et al., 2019; Barba et al., 2019; Jain et al., 2025). Conversely, the lack of a decreasing trend with height implies in-stem microbial production of CH_4_ and N_2_O (Pitz and Megonigal, 2017; Barba et al., 2021). Stem CO_2_ flux results from the combined processes of xylem-mediated transport and stem respiration. Therefore, irregular vertical patterns may indicate a greater contribution from respiratory processes to the flux (Kunert, 2018; Barba et al., 2021).

Changes in hydrological regimes strongly influence the C and N retention capabilities of subtropical soils and their associated GHG dynamics (Wang et al., 2024; Hao et al., 2025). Given that soil water status exerts a primary influence on stem fluxes (Ranniku et al., 2024; Moisan et al., 2025). In subtropical regions, climate change will primarily alter hydrological regimes through more extreme droughts and rainfall (IPCC, 2021), leading to reduced groundwater recharge and increased water stress. A recent study demonstrated that the moisture content of tree stems plays a detrimental critical role in CH_4_, N_2_O and CO_2_ fluxes within wetland and upland forest stems (Ranniku et al., 2024; Moisan et al., 2025). It is well documented that the moisture of stems has a similar impact on tree GHG fluxes among different tree species and at the ecosystem level (Jeffrey et al., 2023; Mander et al., 2022; Ranniku et al., 2024). However, there is scant information on the comparative responses of different stem types (live vs. standing dead trees) at various heights (Hettwer et al., 2025a). In addition, changes in precipitation patterns could lead to more frequent and severe droughts, subsequently lowering groundwater levels. Thus, the interplay of these factors can reshape the hydrological cycling and greenhouse gas dynamics in subtropical forests.

Advancing the understanding of ecosystem-level GHG dynamics under climate change requires further investigation into these processes. A key focus is quantifying how stem fluxes contribute to total forest ecosystem fluxes across varying hydrologic conditions. This study investigated the interannual variability of carbon dioxide (CO_2_), nitrous oxide (N_2_O), and methane (CH_4_) fluxes in a subtropical forest. To elucidate the drivers of tree stem fluxes, we conducted simultaneous measurements of stem and soil fluxes and analyzed their correlations with soil properties and sap flow dynamics. We hypothesized that (1) greenhouse gas fluxes from tree stems significantly decrease during seasonal drought periods and (2) their contribution to the total ecosystem flux significantly increases under humid conditions, and (3) this process is further regulated by the dynamics of tree sap flow.

## Methods

### Study area and design

The field experiment measuring CH_4_ emissions was conducted at Lushan Mountain (29°31′~29°41′ N, 115°51′~116°07′ E) in Jiangxi Province, a subtropical monsoon climate region of China characterized by four distinct seasons (Wu et al., 2018a). The annual average precipitation range of the study area is 1308 to 2068 millimeters, and the annual temperature range is 11.6 to 17.1 °C (Wu et al., 2018b, Wu et al., 2019a). Our previous research has demonstrated forest types at different altitudes in the study area (Wu et al., 2020, Wu et al., 2024). The soil type in this study area was haplic alisols. **Table 1** displays the characteristics of the forest stands included in this study. Twelve representative monitoring points were established for each of the three precipitation treatments (precipitation -80%, precipitation -30% and control) using rain shelters to manipulate throughfall, which located in three 50 × 50 m study plot within the total study area. Each point consisted of two *Cunninghamia lanceolata* live and standing dead trees (dead trees that remain upright in a standing position) with stem chamber installed and one soil chamber.

**Table 1:** Tree stand characteristics.

Tree species	Tree count (n/ha^-1^)	Average tree height (m)	Average tree stem DBH	Canopy coverage
*Cunninghamia lanceolata*	825 ± 98	12.4 ± 2.1	26.8 ± 3.3	0.9 ± 0.08

DBH denotes diameter at breast height (1.3 m).

### Measurement and analysis of stem and soil greenhouse gases

From January 2023 to December 2024, gas samples were collected monthly from static stem chambers installed on *Cunninghamia lanceolata* trees (N = 12), resulting in a total of 24 sampling campaigns. Two measurement chambers per height profile were positioned randomly over a 180^°^circumference, covering a total stem surface area was 0.0108 m^2^ and enclosing a volume for 0.00119 m^3^ (Machacova et al., 2016). Studied stem chambers were fabricated from plastic containers with transparent rectangular (Lock & Lock, Seoul, South Korea) by removing their bases and attaching them to the stem using a neoprene band and hot glue. For gas sampling, the chambers were equipped with detachable lids that provided an airtight seal. To quantify the vertical profile of tree stem fluxes, chambers were positioned at 10 cm, 50 cm, and 150 cm above the ground on smooth stem sections. Sampling was conducted between 09:00 and 13:00. From January 2023 to December 2024, soil gas fluxes were monitored using twelve chambers (0.16 m^2^ area, 0.032 m^3^ volume). Gas samples were collected hourly from each chamber into pre-evacuated (0.3 bar) vials, with four 25 mL mixed samples obtained per sampling event. Concentrations of CO_2_, CH_4_, and N_2_O were quantified using a gas chromatograph (GC-2014, Shimadzu) equipped with electron capture detectors and flame ionization.

### Calculating fluxes and verifying data quality

Flux rates of CO_2_, CH_4_, and N_2_O from stems and soil were determined by linearly regressing the change in chamber headspace gas concentration over time, using Equation 1:

(1) $$F=\frac{M\times P\times V\times \sigma v}{R\times T\times t\times A\times f^{1}}$$

where *F* is the gas flux rate (μg C or N m^-2^ h^-1^), *M* is the molecular mass (44 for CO_2_ and N_2_O, 16 for CH_4_ g mol^-1^), *P* is air pressure (with 101,300 Pa), *V* is volume of chamber (0.00119 m^3^ for stem, 0.032 m^3^ for soil); σv is the slope of the change of gas concentration over time (ppm v), *R* is the gas constant (with 8.314 m^3^ Pa K^-1^ mol^-1^), *T* is laboratory temperature (293.15 K), t is time (1 h), *A* is the surface area covered by the chamber (0.0108 m^2^ for stem, 0.16 m^2^ for soil), and f^1^ is the element-to-compound ratio.

A key quality criterion for the manual chamber measurements was the adjusted R^2^ value from the CO_2_ concentration regression, which verified the integrity of chamber sealing. A threshold R^2^ > 0.9 was applied for flux acceptance; consequently, all stem flux measurements were retained. This resulted in the exclusion of the data, representing 0.11% of CH_4_, 0.04% of N_2_O, and 0.03% of CO_2_ fluxes. Stem CH_4_ and N_2_O fluxes (mean of three heights) data were finally upscaled to a per-hectare basis using stand inventory data (**Table 1**) and established cylindrical tree models (Jeffrey et al., 2019) to assess their ecosystem-level contributions relative to soil fluxes.

### Environmental variables

Throughout the study, soil/air temperature (SoT/AT), water table depth (WTD), and soil water content (SWC) were continuously monitored at each chamber. Soil chambers were fitted with vertically installed temperature (Campbell Sci. 107 probe) and moisture (Delta-T Devices ML3 ThetaProbe) sensors at 0.1 m depth, while WTD was recorded in groundwater wells were using automated loggers (Onset Hobo U20L-04).

### Soil physicochemical properties

Soil samples were collected from 12 points at three depths (0–10, 10–20, and 20–40 cm) between 2023 and 2024, with three cores composited per depth per point, yielding a total of 288 samples. Analyses included: available K^+^ and P (NH_4_-lactate extraction, determined by flame photometry/flow injection); Mg^2+^ and Ca^2+^ (ammonium-acetate extraction, determined by titanium yellow colorimetry/flame photometry); NO_3_^--^N and NH_4_^+^-N (2 mol/L KCl extraction, flow injection); total N (dry combustion via a varioMAX CNS analyzer); soil organic matter (SOM by loss on ignition at 550 °C); and pH (1 M KCl extraction). Additionally, the N_2_ flux potential was assessed via He–O incubation of *ex-situ* cores following established methods (Mander et al., 2014; Butterbach-Bahl et al., 2002).

### Xylem sap flux

Over a two-year period (2023–2024), sap flow was monitored in 12 *Cunninghamia lanceolata* trees using EMS81 systems (EMS Brno, Czech Republic). Sensors were installed 2~2.5 m above ground. Data were recorded at 1-minute intervals and stored as 10-minute averages, directly capturing sap flow and stem temperature (measured under a gauge weather shield). Sap flux density (g h^-1^ cm^-2^) was derived as a key metric, calculated by dividing the sap flow rate (kg/h) by the xylem area (per cm^2^) at the sensor height.

### Statistical analysis

All analyses were performed in R (v4.0.3). As flux data violated normality (Kolmogorov-Smirnov test), non-parametric tests were applied: Kruskal–Wallis with Dunn’s *post hoc* (Bonferroni-corrected) assessed temporal variability and stem flux differences (by type, height, period); Associations between sap flow density and CH_4_, N_2_O, and CO_2_ fluxes from live stems at three heights were examined using Spearman’s rank correlation and visualized via ordinary least squares regression. Data are reported as mean ± SE, with significance at p< 0.05.

## Results

### Temporal variation in stem fluxes

**Figure 1:**
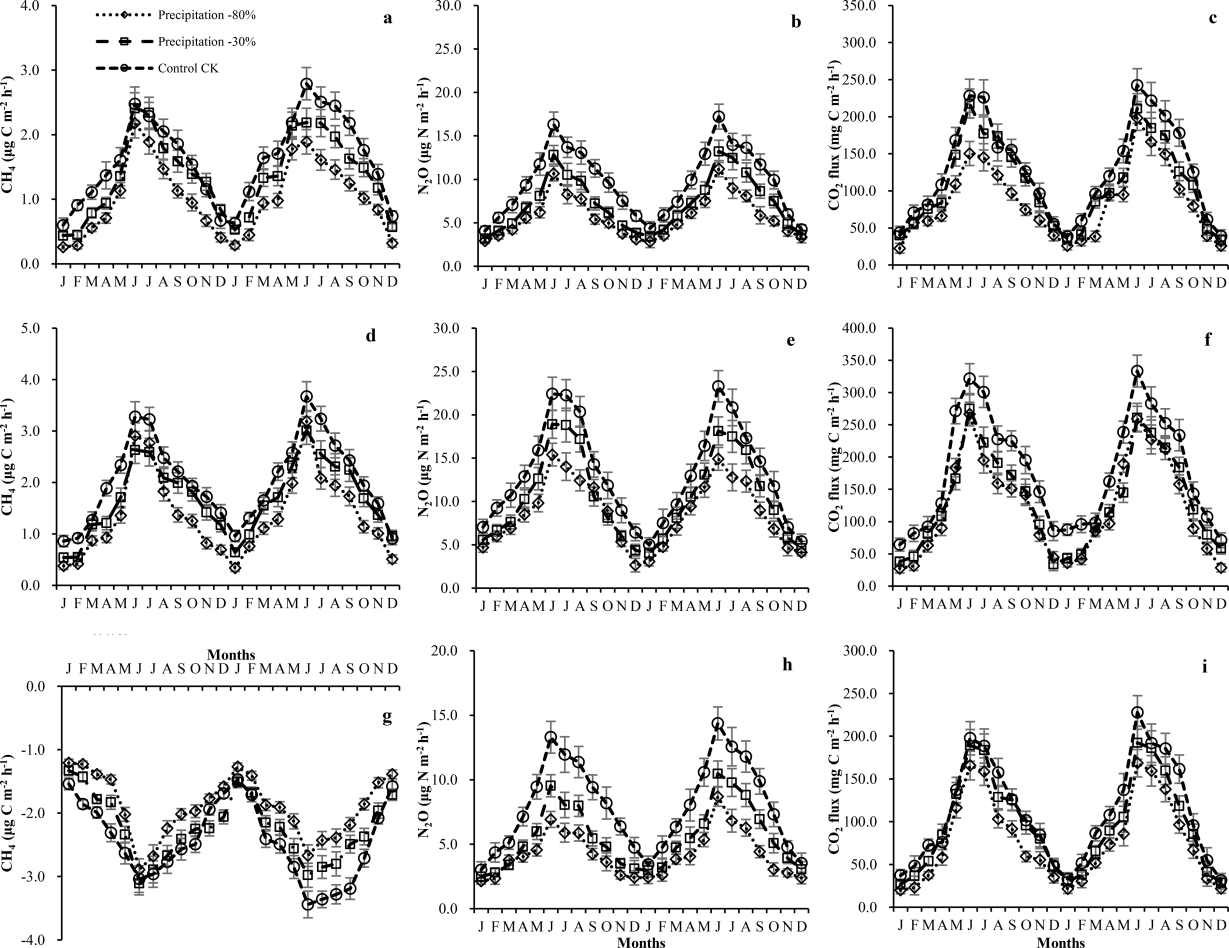
Temporal dynamics of **(a-c)** CH_4_, **(d-f)** N_2_O, and **(g-i)** CO_2_ fluxes from soil and stems alongside environmental parameters. Data shown as mean ± SE (shaded area); fluxes are normalized per m² bark (stem) or ground (soil) area. Units: CH_4_ (μg C m^-2^ h^-1^), N_2_O (μg N m^-2^ h^-1^), CO_2_ (mg C m^-2^ h^-1^).

**Figure 2:**
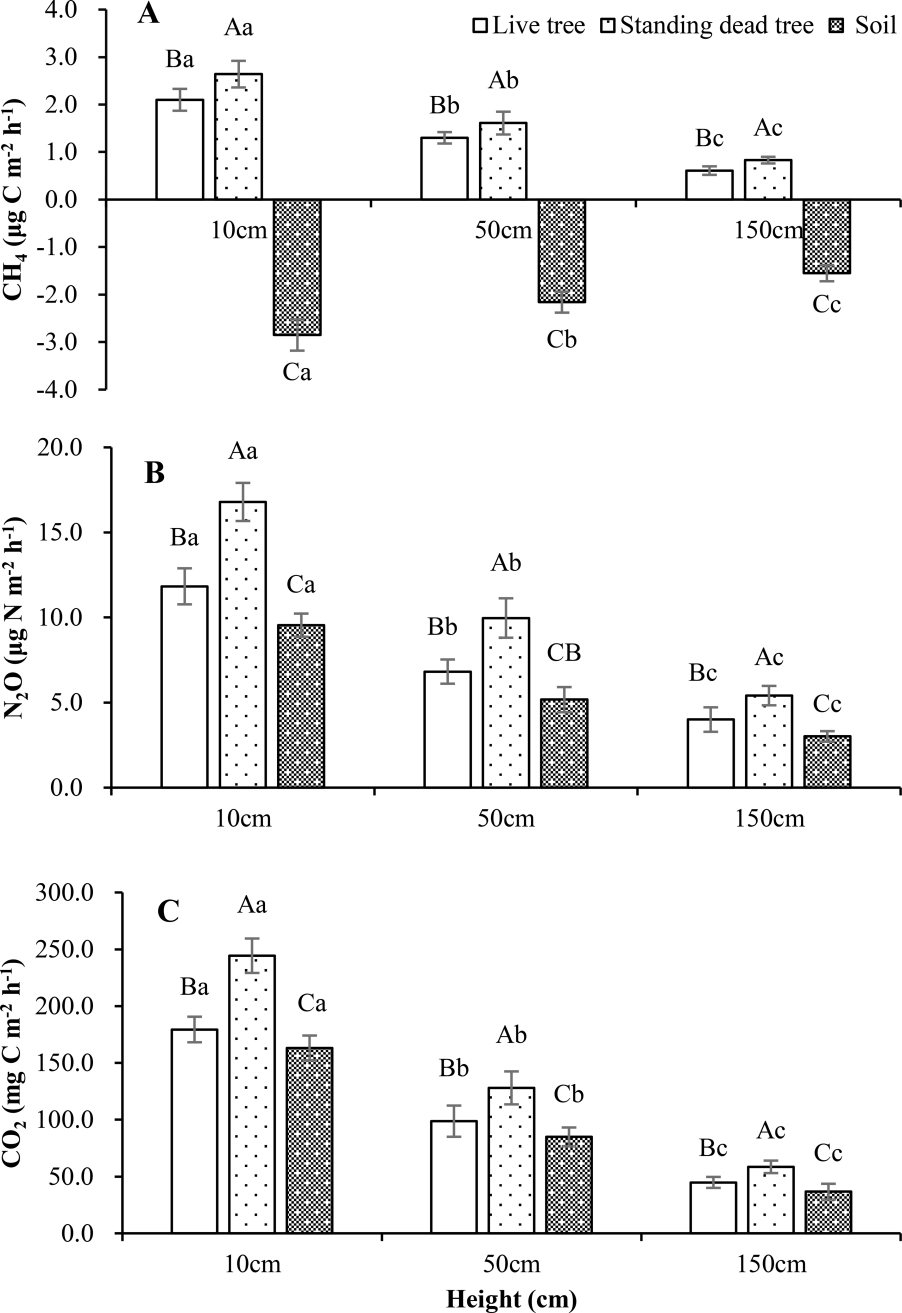
Vertical profiles of CH_4_**(A)**, N_2_O **(B)**, and CO_2_**(C)** stem fluxes measured at 10, 50, and 150 cm heights (N = 144 per height). Lowercase letters indicate significant differences across heights within a source; uppercase letters indicate differences between sources at the same height (Kruskal-Wallis test with *post-hoc* Dunn’s test, p< 0.05).

**Figure 3:**
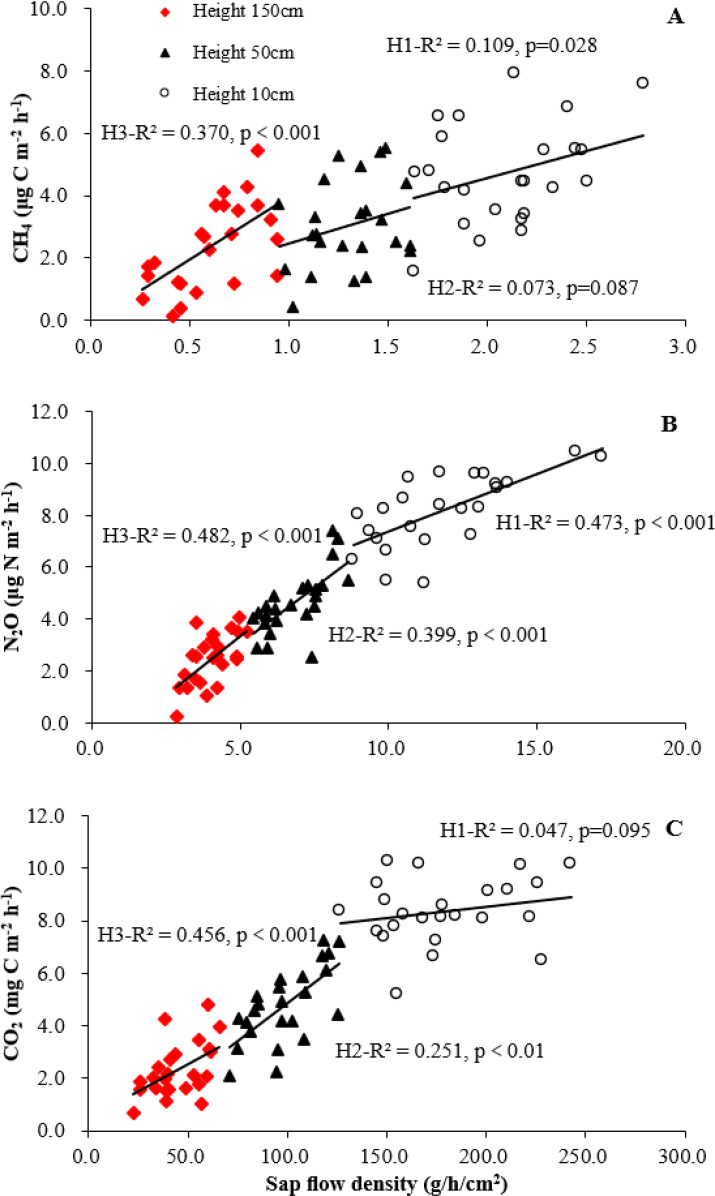
Correlation of sap flow density with live tree stem CH_4_**(A)**, N_2_O **(B)**, and CO_2_**(C)** fluxes at three heights (H1: 10 cm, H2: 50 cm, H3: 150 cm; N = 72). Goodness-of-fit (adjusted R²) and significance (p-value) are derived from linear regression models.

Under all precipitation treatments, tree stems of both types and all heights acted as net annual sources of CH_4_. Regardless of stem types or tree heights, the average CH_4_ fluxes were higher in control treatment than that of precipitation -30% or -80%. Standing dead trees exhibited higher average CH_4_ emissions (2.03 ± 0.23 μg C m^-2^ h^-1^) than live trees (1.62 ± 0.19 μg C m^-2^ h^-1^) under control treatment. Standing dead trees emitted 1.68 ± 0.14 and 1.36 ± 0.15 μg C m^-2^ h^-1^ and live trees emitted 1.37 ± 0.16 and 1.02 ± 0.09 μg C m^-2^ h^-1^ for precipitation -30% and -80% treatments, respectively. **Table 2** displays the average fluxes recorded throughout the study, detailing variations across precipitation treatments and stem types. The temporal dynamics of soil and tree stem CH_4_, N_2_O, CO_2_ fluxes showed similar fluctuations between different precipitation treatments throughout the studied period (**Figure 1**). An increase in both live and standing dead tree stems emissions for different precipitation treatments and peaks occurred in June and July (monthly means up to 1.61 ± 0.16 ~ 2.79 ± 0.25 μg C m^-2^ h^-1^ for live tree and 2.08 ± 0.21 ~ 3.67 ± 0.29 μg C m^-2^ h^-1^ for standing dead tree stems). Statistically significant vertical patterns in stem CH_4_ fluxes were detected, regardless of stem type or precipitation treatment (**Figure 2A**). **Table 3** displays the correlations of the fluxes with relevant meteorological and soil chemical parameters. Both live and standing dead tree stem CH_4_ fluxes exhibited positive correlations with soil temperature (r = 0.23, 0.32), SWC (r = 0.47, 0.54), and WTD (r = 0.19, 0.28) across the study duration. However, live and standing dead tree stem fluxes correlated with soil temperature (*r* = 0.29 and 0.34), stem temperature (*r* = 0.31 and 0.39) and sap flow density (r= 0.40 and 0.55) during the study period (**Table 3**, **Figure 3**).

**Table 2:** Average fluxes (mean ± SE) of CH_4_, N_2_O, and CO_2_ from live stems, standing dead stems, and soil over the full study period (2022–2023).

Treatments	Sources	CH_4_ (μg C m^-2^ h^-1^)	N_2_O (μg N m^-2^ h^-1^)	CO_2_ (mg C m^-2^ h^-1^)
Precipitation -80%	Live tree	1.02 ± 0.09	5.76 ± 0.52	90.5 ± 9.5
	Standing dead tree	1.36 ± 0.15	8.62 ± 0.97	121.5 ± 11.4
	Soil	-1.90 ± 0.11	4.21 ± 0.46	77.9 ± 8.1
Precipitation -30%	Live tree	1.37 ± 0.16	7.21 ± 0.82	111.7 ± 12.4
	Standing dead tree	1.68 ± 0.14	10.46 ± 1.21	132.2 ± 15.7
	Soil	-2.23 ± 0.11	5.53 ± 0.61	96.9 ± 10.3
Control	Live tree	1.62 ± 0.19	9.69 ± 0.99	131.5 ± 13.8
	Standing dead tree	2.03 ± 0.23	13.09 ± 1.37	177.0 ± 15.5
	Soil	-2.43 ± 0.16	7.99 ± 0.83	110.0 ± 12.1

Units: μg C m^-2^ h^-1^ (CH_4_), μg N m^-2^ h^-1^ (N_2_O), mg C m^-2^ h^-1^ (CO_2_).

**Table 3:** Spearman correlations between stem/soil GHG fluxes and topsoil (0~10 cm) environmental/chemical parameters.

Index		CH_4_ (μg C m^-2^ h^-1^)			N_2_O (μg N m^-2^ h^-1^)			CO_2_ (mg C m^-2^ h^-1^)		
		Liv tree	StD tree	Soil	Liv tree	StD tree	Soil	Liv tree	StD tree	Soil
SWC (m^3^m^-3^)		0.47^*^	0.54^**^	0.69^**^	0.43^*^	0.46^*^	-0.36^*^	-0.45^*^	-0.47^*^	-0.61^**^
WTD (cm)		0.19	0.28	0.45^*^	0.36^*^	0.47^*^	-0.05	-0.44^*^	-0.49^*^	-0.57^**^
SoT (°C)		0.23	0.32^*^	-0.53^**^	-0.38^*^	-0.42^*^	0.27	0.76^***^	0.84^***^	0.87^***^
AT (°C)		0.29	0.34^*^	-0.49^*^	-0.43^*^	-0.48^*^	0.31^*^	0.91^***^	0.95^***^	0.89^***^
StT (°C)		0.31^*^	0.39^*^	0.17	0.25	0.29	0.22	0.73^***^	0.78^***^	0.71^***^
SFD (g/h/cm^2^)		0.40^*^	0.55^**^	–	0.87^***^	0.81^***^	–	0.80^***^	0.79^***^	–
Soil	CH_4_ (μg C m^-2^ h^-1^)	-0.95^***^	-0.89^***^	**1**	-0.86^***^	-0.81^***^	-0.83^***^	-0.88^***^	-0.82^***^	-0.88^***^
	N_2_O (μg N m^-2^ h^-1^)	0.82^***^	0.84^***^	-0.83^***^	0.98^***^	0.90^***^	**1**	0.85^***^	0.84^***^	0.82^***^
	CO_2_ (mg C m^-2^ h^-1^)	0.87^***^	0.89^***^	-0.88^***^	0.87^***^	0.88^***^	0.82^***^	0.96^***^	0.93^***^	**1**

Significant correlations (p< 0.05) are in bold. * *p* < 0.05, ** *p* < 0.01, *** *p* < 0.001. Abbreviations: Liv, living tree; StD, standing dead tree; SWC, Soil water content; WTD, Water table depth; SoT, Soil temperature; AT, Air temperature; StT, Stem temperature; SFD, Sap flow density.

Live and standing dead tree stems were an annual average emitter of N_2_O under control, precipitation -30% and -80% treatments were 9.69 ± 0.99, 7.21 ± 0.82, 5.76 ± 0.52 and 13.09 ± 1.37, 10.46 ± 1.21, 8.62 ± 0.97, respectively (**Table 2**). Live and standing dead tree stem fluxes perform a similar release and pronounced temporal variability under different precipitation treatment throughout the studied period (**Figures 1b-e**). The largest emissions for different precipitation treatments occurred in June and July, both in 2023 and 2024, with maximum values of 17.22 ± 1.43 and 23.29 ± 1.81 μg N m^-2^ h^-1^ for live and standing dead tree stem in July 2024, respectively. Tree stem N_2_O fluxes exhibited a significant negative correlation with stem height (**Figure 2B**). During this study period, mean fluxes of live and standing dead tree stem were 11.82 ± 1.06, 16.79 ± 1.12 μg N m^-2^ h^-1^ at height 10 cm, 6.82 ± 0.71, 9.97 ± 1.16 μg N m^-2^ h^-1^ at height 50 cm and 4.00 ± 0.72, 5.41 ± 0.57 μg N m^-2^ h^-1^ at height 150 cm, respectively. Both live and standing dead tree stems N_2_O fluxes showed a positive relationship with WTD (*r* = 0.36 and 0.47), SWC (*r* = 0.43 and 0.46), stem temperature (*r* = 0.25 and 0.29) and sap flow density (r= 0.87 and 0.81), but negative correlations with soil (*r* = -0.38 and -0.42) and air temperatures (*r* = -0.43 and -0.48) (**Table 3**, **Figure 3**).

Throughout the study, both live and standing dead trees acted as net sources of CO_2_, with an average of 131.5 ± 13.8, 111.7 ± 12.4, 90.5 ± 9.5 mg C m^-2^ h^-1^ for live tree and 177.0 ± 15.5, 132.2 ± 15.7, 121.5 ± 11.4 mg C m^-2^ h^-1^ for standing dead tree for control, precipitation -30% and -80%, respectively (**Table 2**). A reduction in monthly average fluxes was observed for both stem types during winter season (January and February) (**Figures 1c-f**). Fluxes peaked in the summer months, the maximum peak occurs in June and July, with monthly averages reaching 333.4 ± 24.7, 242.6 ± 22.2 mg C m^-2^ h^-1^ (standing dead tree and live tree), 275.3 ± 21.3, 217.8 ± 19.6 mg C m^-2^ h^-1^ and 266.8 ± 18.3, 198.4 ± 16.8 mg C m^-2^ h^-1^ for control, precipitation -30% and -80%, respectively (**Figures 1c-f**). Both live and standing dead tree stems exhibited a vertical trend in CO_2_ fluxes, with the highest values at the base (179.4 ± 11.24 and 244.3 ± 15.17 mg C m^-2^ h^-1^, respectively) that progressively decreased at 50 cm (to 98.7 ± 13.73 and 128.0 ± 14.49 mg C m^-2^ h^-1^) and 150 cm (to 44.8 ± 4.84 and 58.5 ± 5.51 mg C m^-2^ h^-1^) (**Figure 2C**). The CO_2_ fluxes were significantly higher in tree height 10 cm than that of 50 cm and 150 cm (**Figure 2C**). Live and standing dead tree stem CO_2_ efflux exhibited strong correlated positively with soil (*r* = 0.76 and *r* = 0.84) and air temperatures (*r* = 0.91 for live tree and *r* = 0.95 for standing dead tree), and stem temperatures (*r* = 0.73 and 0.78) (**Table 3**). Live and standing dead tree stem CO_2_ fluxes exhibited significant correlations with WTD (*r* = -0.44 and -0.49), SWC (*r* = -0.45 and -0.47) and sap flow density (r= 0.80 and 0.79) (**Table 3**, **Figure 3**).

### Temporal variation in soil fluxes

The soil at the study site acted as a net CH_4_ sink (-2.43 ± 0.16, -2.23 ± 0.11 and -1.90 ± 0.11 μg C m^-2^ h^-1^ for control, precipitation -30% and -80% treatments) from the atmosphere, and a source of N_2_O (7.99 ± 0.83, 5.53 ± 0.61 and 4.21 ± 0.46 μg N m^-2^ h^-1^) and CO_2_ (110.0 ± 12.1, 96.9 ± 10.3 and 77.9 ± 8.1 mg C m^-2^ h^-1^) for different precipitation treatments during the experimental period. Soil CH_4_ fluxes exhibited distinct monthly dynamics, with peak summer uptake (June–July) reaching -3.44 ± 0.21 to -2.43 ± 0.14 μg C m^-2^ h^-1^ (**Figure 1g**). Average soil fluxes for the studied periods under different stem types (live and standing dead tree) and precipitation treatments (control, precipitation -30% and -80%) are presented in **Table 2**. Soil CH_4_ fluxes correlated positively with SWC (*r* = 0.69) and WTD (*r* = 0.45), and negative correlations soil (*r* = - 0.53) and air temperature (*r* = - 0.49) (**Table 3**).

Soil N_2_O fluxes showed episodic winter and summer peaks, reaching 5.89 ± 0.73 to 14.36 ± 1.28μg N m^-2^ h^-1^ in June–July, and were positively correlated with air (r = 0.31) and soil (r = 0.27) temperature (**Table 3**).

A seasonal trend was observed in soil CO_2_ fluxes during the studied period (**Figure 1i**). An increase in emissions was observed through spring, peaking in June–July (max monthly average: 158.6 ± 16.1 ~ 227.8 ± 19.7 mg C m^-2^ h^-1^) prior to a decline in late summer. Soil CO_2_ fluxes correlated positively with soil (r=0.87) and air (r=0.89) temperatures, and negatively with SWC (r=-0.61) and WTD (r=-0.57). A significant positive correlation was observed between stem and soil fluxes for CH_4_ (r > 0.80), N_2_O (r > 0.82), and CO_2_ (r > 0.82) in both live and standing dead trees (**Table 3**).

### Contribution of soil and stem fluxes to total ecosystem fluxes

**Figure 4:**
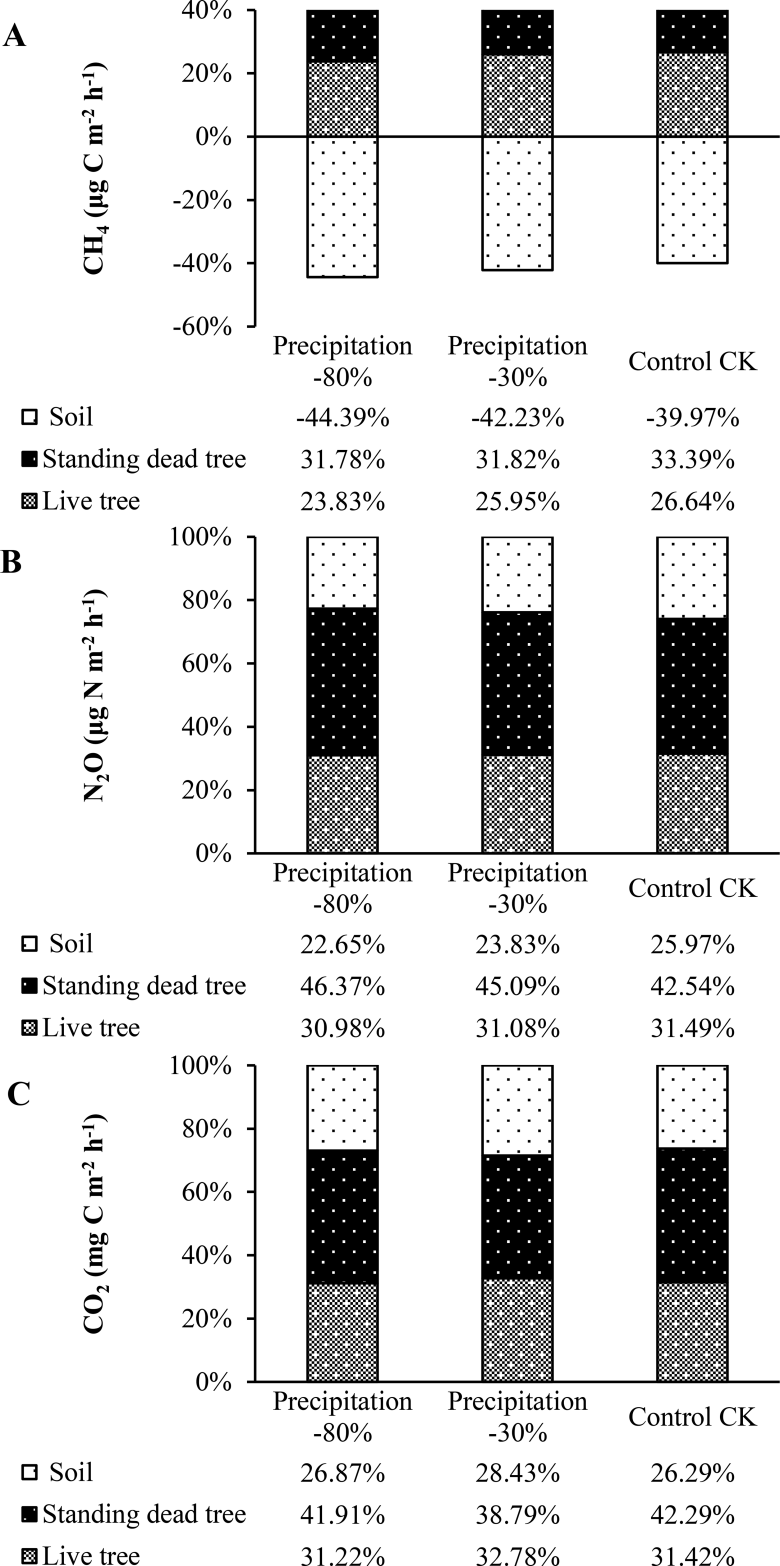
**(A)** CH_4_, **(B)** N_2_O, and **(C)** CO_2_ flux contributions from stems (live and dead trees) and soil, expressed per unit forest ground area, with positive and negative values indicating emission and uptake.

The source contribution of soil and stem to total fluxes was quantified (**Figure 4**). Throughout the measurement period, upscaled stem CH_4_ release from live and standing dead trees offset the soil CH_4_ sink by 13.33%, 16.28%, 20.56% and 6.58%, 10.41%, 12.61% under control, -30%, and -80% precipitation treatments, respectively (**Figure 4A**). The proportional contribution of stem CH_4_ release (live and standing dead trees combined) relative to soil emissions was 1.37, 1.25, and 1.50 under control, -30%, and -80% precipitation treatments, respectively (**Figure 4A**). Stem N_2_O fluxes from both stem types (live and standing dead tree) accounted for 1.85, 2.20 and 2.42 times of the combined soil emissions for control, precipitation -30% and -80%, respectively. In addition, the N_2_O fluxes of standing dead tree stems were 1.35, 1.45 and 1.50 higher than that of live tree stems. The proportional contribution of stem N_2_O release (live and standing dead trees combined) relative to soil emissions was 3.20, 3.42, and 2.85 under control, -30%, and -80% precipitation treatments, respectively (**Figure 4B**). Stem CO_2_ fluxes respectively shown under control (live tree for 31.42% and standing dead tree for 42.29%), precipitation -30% (32.78% and 38.79%) and precipitation -80% (31.22% and 41.91%) of total stem and soil CO_2_ release, while the soil added 26.29%, 28.43% and 26.87% for control, precipitation -30% and -80%, respectively (**Figure 4C**). The proportional contribution of stem CO_2_ release (live and standing dead trees combined) relative to soil emissions was 2.52, 2.72, and 2.80 under control, -30%, and -80% precipitation treatments, respectively (**Figure 4C**).

The fluxes of all three gases varied significantly across months throughout the study period. CH_4_ flux partitioning exhibited strong seasonal variations, particularly between summer (June, July) and winter (December, January) months. Compared with control treatment, stem and soil CH_4_ emissions under precipitation -30% and -80% treatments were decreased, and live and standing dead tree stem fluxes resulted in a 60.03%, 57.77% and 55.61% offset of the soil sink under control, precipitation -30% and -80% treatments (**Figure 4A**). The contribution of tree stem N_2_O fluxes to the total stem flux and soil was higher in standing dead tree stem than live tree for different precipitation treatments during the studied periods. For the standing dead tree stem, combined stem fluxes were relatively sources of 42.54%, 45.09% and 46.37% for control, precipitation -30% and -80% to total soil and stem N_2_O, whereas for the live tree stem, stem fluxes combined decreased by 25.97%, 23.83% and 22.65% under different precipitation treatments to total soil and stem emissions (**Figure 4B**). Although net CO_2_ emissions were elevated during the study period, compared to other treatments, control conditions resulted in a higher contribution from stem fluxes (higher 22.80% and 44.36% than precipitation -30% and -80% treatments, respectively), with standing dead tree stem fluxes contributing a 1.35, 1.18, 1.34 times higher than live tree fluxes (**Figure 4C**). Live and standing dead tree stem fluxes accounting for 31.22~32.78% and 38.79~42.29% of total CO_2_ release of the ecosystem under different precipitation treatments, respectively (**Figure 4C**). Soil fluxes thus accounted for 26.29%, 28.43% and 26.87% for control, precipitation -30% and -80% of the total soil and stem CO_2_ fluxes during the studied periods, respectively (**Figure 4C**).

## Discussion

### Changes of stem CH_4_

Previous studies on various broadleaved species reported higher annual CH_4_ release rates than our findings for live and standing dead trees. Compared to our site, the higher soil moisture reported in floodplain forests, riparian zones, peatlands, and forested wetlands (e.g., Moldaschl et al., 2021; Mander et al., 2022; Ranniku et al., 2024; Jeffrey et al., 2023; Pangala et al., 2015) likely explains this discrepancy. Long-term measurements across forests with varying precipitation regimes are currently scarce, limiting the contextualization of our subtropical findings. Higher SWC and WTD promote anaerobic conditions for soil methanogenesis, thus increasing CH_4_ availability for uptake by tree roots or tree conduits (Barba et al., 2019a). Throughout the study, soil hydrological conditions and temperature were the primary drivers of both soil and stem CH_4_ fluxes (**Table 3**), which support our first hypothesis. Their strong correlation further suggests a shared hydrological connection. However, this study is the first to document sap flow relationships with stem CH_4_, N_2_O, and CO_2_ fluxes in both live and standing dead trees under differing precipitation regimes. The efficiency of sap flow in driving stem gas efflux depends on the initial concentration of the dissolved gas. Further investigation is warranted to elucidate the mechanisms of upward gas transport from roots and the differences between live and dead trees.

Production of CH_4_ occurs both in the soil and directly within or on tree stems. There is no consensus on the exact contribution of these CH_4_ sources (Pitz and Megonigal, 2017; Barba et al., 2019a; Jeffrey et al., 2021). Our findings support a combined contribution of soil and stem sources to the net CH_4_ flux in live and standing dead trees, regardless of precipitation treatment, which support our second hypothesis. The consistent correlation of stem CH_4_ fluxes with soil parameters across treatments (**Table 3**) points to a soil linkage, yet the lack of sap flow influence (**Figure 3A**) and the declining vertical flux profile (**Figure 2A**) suggest *in-situ* microbial production (especially in dead trees) as a concurrent source. This is corroborated by stem emissions occurring despite soil being a net CH_4_ sink, implying a deep anaerobic origin (Ranniku et al., 2023; Machacova et al., 2023; Pitz et al., 2018). Temporally, emission peaks in June–July for both species tracked increases in SWC (**Table 3**). The combination of sustained high-water levels and increasing soil temperatures in early summer was likely pivotal in creating optimal conditions for methanogenesis, corresponding with a more pronounced birch emission peak (Pangala et al., 2015). In this study, overall, stem CH_4_ emissions remained low under the imposed drought conditions, which support our first hypothesis. The observed fluxes were primarily driven by transient peaks, occurring at precipitation levels -30% and -80% treatments. The peak emissions observed under wetter conditions (control *vs*. precipitation -30% and -80%) indicate that baseline stem CH_4_ is a mixture of stem and soil-derived fluxes, which support our third hypothesis. However, the emission peaks are driven primarily by soil hydrological changes, implying that pulses of soil-originated CH_4_ are responsible for these events across both live and standing dead trees.

### Changes of stem N_2_O

*Cunninghamia lanceolata* stems showed higher annual N_2_O fluxes than birch (Machacova et al., 2019), spruce (Ranniku et al., 2024), alder (Mander et al., 2021), ash and poplar (Moldaschl et al., 2021) reported in other forest ecosystems. Stem N_2_O fluxes peaked in summer (June–July; **Figures 1b-e**), governed by SWC and WTD changes. These stem peaks temporally coupled with soil N_2_O emissions, forming a hot moment driven by subsurface gas transport (Ranniku et al., 2024). Elevated SWC likely increased soil NO_3_^--^N availability, enhancing denitrification under anaerobic conditions and triggering the observed N_2_O peaks. Reduced precipitation depleted the soil N pool, suppressing denitrification and decreasing N_2_O fluxes (**Figures 1b-e**). However, monthly sampling likely missed rapid N dynamics that could better explain stem fluxes. The observed short-lived peaks confirm that both live and dead stems respond rapidly to hydrological shifts, particularly during wet-dry cycles (Ranniku et al., 2023, Ranniku et al., 2024). Higher-frequency measurements are needed to accurately capture emission peaks and improve estimates of stem flux contributions to annual forest GHG budgets, especially across varying stem types and precipitation regimes (Barba et al., 2019b; Barton et al., 2015).

The apparent inverse correlation between stem N_2_O fluxes and temperature likely arose because summer peak emissions coincided with temperature-enhanced denitrification, which concurrently stimulated nitrification and N_2_O production. Therefore, stem N_2_O fluxes are primarily driven by changes in soil water status rather than by temperature dynamics under differing precipitation conditions, which support our first and second hypothesis. The dominant soil origin of N_2_O from live stems was supported by strong correlations with soil parameters and sap flow, along with a decreasing vertical flux trend, which supports our third hypothesis. In contrast, emissions from standing dead stems were likely attributable to both soil conduction and *in situ* production within the stems.

### Changes of stem CO_2_

Our measured net annual CO_2_ fluxes from live and standing dead stems substantially exceeded those reported for European beech (Machacova et al., 2023) and Scots pine (Kolari et al., 2009) (by approximately 12-fold), and were moderately higher (by ~1.7-fold on average) than values for Birch and Spruce in northern peatlands (Ranniku et al., 2024). Soil and tree stem CO_2_ fluxes peaked in summer and declined in winter, showing consistent seasonal dynamics (**Figures 1a-c**). A close coupling between stem flux trends and growth phenology appears to be a common phenomenon in various forest ecosystems. This has been documented in species including Norway spruce (subalpine; Etzold et al., 2013), Scots pine (boreal; Kolari et al., 2009), European beech (temperate uplands; Machacova et al., 2023), and Birch/Spruce (northern peatlands; Ranniku et al., 2024). Temperature was the primary driver of CO_2_ efflux, with both stem and soil releases showing elevated levels during the summer, a pattern consistent with the dynamics observed for CH_4_ and N_2_O (**Table 3**). This finding aligns with prior work, confirming it as a recognized effect (Takahashi et al., 2022; Ojanen et al., 2010; Barba et al., 2019). The underlying mechanism involves temperature-sensitive respiration and diffusion in these ecosystems (Teskey et al., 2008; Ranniku et al., 2024). Higher CO_2_ efflux in drier periods is a consequence of reduced transpiration, resulting in increased concentrations of CO_2_ gas inside the stem (Salomón et al., 2016). Elevated stem water content under wetter conditions impedes radial diffusion, thereby explaining the observed negative correlation with soil water parameters (Salomón et al., 2016; Gansert and Burgdorf, 2005; Bowman et al., 2005). Stem-emitted CO_2_ originates from root and stem respiration, with a minor contribution from root uptake of dissolved CO_2_ in soil water (Bloemen et al., 2013; Aubrey and Teskey, 2009). Therefore, decreased precipitation could decrease the CO_2_ conduction of root and stem for live and standing dead trees, which support our first and second hypothesis. However, the origin of stem CO_2_ efflux from different tree types (live vs. standing dead trees) remains debated owing to the complexity of its components. A likely difference in the primary driver of stem CO_2_ efflux was identified: root uptake for live trees versus root conduction for standing dead trees.

In this study, elevated CO_2_ fluxes from the lower stem, together with their vertical profile (**Figure 2C**) and significant correlations with xylem sap flow (**Figure 3C**), support our third hypothesis and demonstrated stem CO_2_ emissions are primarily derived from soil-transported CO_2_. These findings align with previous reports of pronounced vertical gradients in stem CO_2_ efflux (Tarvainen et al., 2014; Mills et al., 2025) and linear relationships between stem CO_2_ efflux and xylem sap CO_2_ concentration (Anttila et al., 2024; Bréchet et al., 2025). However, reported vertical gradients of stem CO_2_ flux are inconsistent, which can be attributed to the dissolution and internal transport of respired CO_2_ away from its production site (Ranniku et al., 2024; Salomón et al., 2024). A key question is whether a direct relationship exists between sap flow and stem CO_2_ efflux separate from the overriding influence of air temperature (Hölttä and Kolari, 2009). Investigating this, especially for standing dead trees, is an important research direction. Further investigation is needed into stem CO_2_ fluxes across the vertical profile, particularly at higher positions. Additionally, the effects of stem and bark anatomy on diffusion rates, and their differences between live and dead trees, must be determined.

### Stem specific effects

The interspecific variation in temporal tree stem flux patterns underscores the need to understand species-specific GHG drivers, as flux differences can originate from microtopography-mediated variations in SWC and WTD. Therefore, the potentially greater water availability to roots in depressions suggests that these trees could be sources of elevated N_2_O and CH_4_ fluxes (Terazawa et al., 2015; Jeffrey et al., 2020). The upward transport of dissolved gases in xylem is governed by spatial variations in soil water gas concentrations (Machacova et al., 2016; Machacova et al., 2019). Furthermore, root activity, depth, and density can exhibit variation across different tree stem types and under varying precipitation conditions. The fine root counseling ability of standing dead trees is generally higher than live trees. Standing dead trees often exhibit enhanced gas transport, particularly of CH_4_. This is particularly evident for CH_4_, as it can be produced in deep soil zones via methanogenesis and readily transported upward (Ranniku et al., 2023; Puhe, 2003). Differences in gas diffusion and conduction rates between live and standing dead trees are driven by contrasts in their stem morphology and physiology (Pitz and Megonigal, 2017; Ranniku et al., 2024). Consequently, standing dead trees, with their typically higher conduction rates, are presumed to be sources of elevated GHG fluxes (unpublished data). A key structural divergence exists between conifers, which utilize tracheid, and standing dead trees that may develop wider vessels, thereby allowing for more efficient root-to-stem gas transport in the latter (Gorgolewski, 2022). The bark characteristics of standing dead trees, in contrast to those of live trees, are more favorable and may facilitate axial gas diffusion through the bark (Martinez and Ardón, 2021; Gorgolewski, 2022). Nevertheless, research remains limited on how precipitation-regulated GHG fluxes in live and standing dead trees are driven by wood and bark anatomy, impeding a mechanistic understanding of gas transport across the atmosphere-tree-soil continuum.

Tree stem emissions can constitute a major portion of the total soil-stem GHG flux. The exclusion of stem fluxes (from both live and standing dead trees) from forest GHG inventories may result in inaccurate budgets for the respective gases (Gorgolewski, 2022; Ranniku et al., 2024; Machacova et al., 2023). Throughout the study, CH_4_ fluxes from live and standing dead stems under control, -30%, and -80% precipitation treatments offset the soil sink by 60.03%, 57.77%, and 55.61%, respectively (**Figure 4A**), which support our second hypothesis. Evidence from temperate upland and northern peatland forests shows that trees can offset the soil CH_4_ sink (Machacova et al., 2023; Ranniku et al., 2024; Pitz and Megonigal, 2017). Conversely, in ecosystems where soils are a CH_4_ source, tree stems amplify the net budget; for example, they contribute an additional 83% to the total CH_4_ flux of soil in some wetland forests (Pangala et al., 2015; Mander et al., 2022; Jeffrey et al., 2023). For the live and standing dead tree stem, stem fluxes were relative sources of about 22.65%~25.97% and 42.54%~46.37% for different precipitation to total soil and stem N_2_O, respectively (**Figure 4B**). Low stem contributions have been documented in a boreal forest (birch 0.75%; spruce 2.5%), a riparian forest (alder 0.8%), and northern peatland forests (birch 3.0%) (Machacova et al., 2019; Mander et al., 2021; Ranniku et al., 2024). A significant portion of the ecosystem’s CO_2_ efflux originated from tree stems, with live and standing dead trees contributing 31.22~32.78% and 38.79~42.29%, respectively, and collectively accounting for the majority of the total release (**Figure 4C**). Although long-term stem CO_2_ flux monitoring is rare, studies report that stems account for 28.4% of fluxes in a temperate upland forest and up to 81% in northern peatlands during the growing season (Warner et al., 2017; Ranniku et al., 2024). However, the extrapolation of chamber-based stem measurements (often from the base) to the tree level constitutes a key uncertainty (Barba et al., 2024) and a potential source of inaccuracy in ecosystem flux quantifications.

A significant seasonal shift was observed in the proportion of total fluxes attributable to stem fluxes, particularly when comparing wetter and warmer periods. Under control conditions, the soil exhibited an enhanced CH_4_ sink in summer, while stem emissions functioned as a source. However, pronounced differences in summer stem CH_4_ emissions and soil uptake were observed, with the control treatment significantly surpassing both the -30% and -80% precipitation scenarios (**Figure 4A**). According to Mander et al. (2022), stem emissions are responsible for shifting riparian forest ecosystems to net CH_4_ sources during wet conditions. Soil hydrological conditions emerged as a key driver of stem CH_4_ dynamics, as evidenced by flux variations across precipitation regimes. Conversely, the contribution of stem N_2_O fluxes remained similar across precipitation treatments, irrespective of whether the stems were live or standing dead tree (**Figure 4B**). The small variance suggests a high responsiveness of stem N_2_O fluxes to short-term experimental hydrological conditions such as precipitation and drought (Mander et al., 2021). Total CO_2_ efflux from stems and soil was elevated under reduced precipitation (-30% and -80%), whereas stems contributed a larger fraction of the total in the control treatment (**Figure 4C**). Given that temperature is a stronger determinant, changes in hydrologic conditions play a secondary role in stem CO_2_ dynamics (Kolari et al., 2009; Etzold et al., 2013).

This study was conducted on a single tree species (*Cunninghamia lanceolata*) at a single site (Lushan Mountain), which limits the direct generalizability of our findings to other subtropical forest ecosystems. Nevertheless, we propose that the substantial contribution of standing dead trees to stem greenhouse gas emissions may be a functionally conserved trait across diverse forest types, given their ubiquitous role as hotspots for gas diffusion pathways. Moreover, similar positive relationships between stem CO_2_ efflux and xylem sap flow have been reported in other temperate and tropical tree species, suggesting that the biophysical mechanisms underlying soil-derived CO_2_ transport may be broadly applicable. Further comparative studies across multiple subtropical species and successional stages are needed to confirm the generality of these patterns.

## Conclusions

In the subtropical forest, standing dead trees contributed more significantly to annual greenhouse gas (GHG) dynamics than live trees. Tree stem CH_4_ and N_2_O fluxes showed sporadic emission peaks, whereas CO_2_ fluxes peaked in summer, aligning with temperature-driven seasonal trends. Hydrological variation primarily regulated CH_4_ and N_2_O fluxes, while temperature mainly influenced CO_2_ fluxes. Additionally, monthly variation, soil water content (SWC), and tree status (live or dead) significantly affected stem GHG fluxes across precipitation treatments.

Stem CH_4_ from both live and dead trees offset 55.61~60.03% of the soil CH_4_ sink under varying precipitation. In contrast, stems acted as net sources of N_2_O and CO_2_, contributing 30.98~46.37% and 31.22~42.29%, respectively, to total emissions. This study is the first to report relationships between sap flow and stem CH_4_, N_2_O, and CO_2_ fluxes in live trees, and to compare these with standing dead trees under different precipitation regimes in a subtropical forest. These relationships—combined with correlations to other measurement environmental factors and vertical GHG flux profile—suggest that net CH_4_ fluxes derive from both soil transport and stem-based production, whereas N_2_O and CO_2_ originate primarily from soils.

Future research should prioritize high-frequency and long-term measurements across diverse stem types, precipitation regimes, and ecosystems to better quantify the contribution of different tree stem fluxes to annual GHG budgets in forests.

## Data Availability

The raw data supporting the conclusions of this article will be made available by the authors, without undue reservation.
